# Ongoing Voluntary Settlement and Independent Agency: Evidence from China

**DOI:** 10.3389/fpsyg.2017.01287

**Published:** 2017-07-27

**Authors:** Jing Feng, Xiaopeng Ren, Xinran Ma

**Affiliations:** ^1^CAS Key Laboratory of Behavioral Science, Institute of Psychology Beijing, China; ^2^University of Chinese Academy of Sciences Beijing, China

**Keywords:** voluntary frontier settlement, independent self, relational mobility, nepotism

## Abstract

Voluntary frontier settlement leads to independent agency. Since this type of research has not yet been implemented in ongoing voluntary settlement frontiers, we conducted several cultural tasks to investigate Shenzhen, known as China’s ongoing “South Frontier,” which is composed mostly of people that have emigrated from other Chinese provinces within the past 30 years. We hypothesized that residents of Shenzhen are more independent than those in other regions of Mainland China. As predicted, residents of Shenzhen scored higher than China inland residents in self-reported independent beliefs and scored lower in nepotism. The results indicate that, even in a short-term ongoing frontier, voluntary settlement is associated with independent agency.

## Introduction

Cross-cultural research indicates that there are obvious behavioral and cultural differences between North American individuals and Eastern Asian individuals (Markus and Kitayama, 1991; Heine, 2001). Due to different cultural backgrounds and history, North Americans and Eastern Asians are different in many aspects such as independent/interdependent agency, relational mobility, interpersonal communication, etc. Many theories and hypotheses have been developed to explain why there are cultural differences in individualism/collectivism or independent/interdependent self-construal (Kitayama et al., 2006; Yuki et al., 2013; Ishii, 2014; Ishii et al., 2014). One of the most famous hypotheses in recent years is the voluntary frontier settlement hypothesis (Kitayama et al., 2006, 2014).

## Voluntary Frontier Settlement Hypothesis

The main idea of the voluntary frontier settlement hypothesis is that regions mainly composed of immigrants that settled voluntarily have some unique cultural features that are likely the result of immigration history (Kitayama et al., 2014). For example, Europeans’ voluntary settlement in North America during the formative years of the United States had an important role in forming American individualism (Bellah et al., 1985; Kitayama et al., 2006). Compared with Western Europeans with the same religious traditions and similar levels of development, Americans are more individualistic in many cultural tasks and cultural products (Kitayama et al., 2009; Varnum and Kitayama, 2011). Within-culture variation in individualism in the United States can also be explained by voluntary frontier settlement (Vandello and Cohen, 1999; Varnum and Kitayama, 2011; Varnum, 2012). Furthermore, there is evidence linking voluntary frontier settlement to individualism in interdependent societies. For example, a few studies have examined Hokkaido, a northern island of Japan with a voluntary settlement history during the 19th century, and these areas showed more independent agency measured by cultural tasks such as implicit social orientation, dissonance reduction, and attribution tasks (Kitayama et al., 2006; Ishii, 2014; Ishii et al., 2014). Researchers have argued that the frontier of the modern world is the cosmopolitan city (Sevincer et al., 2015).

There are three possible mechanisms underlying the establishment of frontier spirit: (1) self-selection for settlement, which means voluntary settlers are self-motivated to move to a new place for personal goals and development; (2) adaptation and acculturation, where challenging situations and environments reinforce settlers’ independent agency; and (3) institutionalization of tacit beliefs and practices of independence, meaning that the culture and social regulation in chosen settlement regions influence future generations and prospective immigrants reciprocally (Kitayama et al., 2006). Two studies explored the relative importance of these three mechanisms toward independent agency (especially self-selection versus adaptation) and found self-selection was more important than adaptation in both geography and its new form-cosmopolitan city (Kitayama et al., 2006; Sevincer et al., 2015).

Yamagishi et al. (2012) reference the German phrase “city air makes you free” in their frontier hypothesis. The “city air” hypothesis posited that cities have fewer social constraints, which frees residents from pressure to suppress their pursuit of individual goals. The city air hypothesis emphasizes that urbanization leads to independent selves. The authors themselves pointed out that this hypothesis is quite broad and does not point to particular aspects of urban social ecology that promote independent agency (Yamagishi et al., 2012). They also suggested that mobility might be another potential factor of the city air hypothesis. Even the concept of mobility is not so straightforward, there are different kinds of mobility, such as relational mobility, vocational mobility, and residential mobility (Chen et al., 2009; Schug et al., 2009; Oishi, 2010). Residential mobility has probably been studied the most in these three types. Residential mobility can be defined as the frequency with which individuals change their places, and it has been linked to independent self-construal (Oishi, 2010, 2014). Although these are separate concepts, they should be correlated. Cultures that tend to have more residential mobility should also have more vocational and relational mobility (Oishi et al., 2015).

Both the voluntary frontier settlement hypothesis and research on mobility support the idea that mobility enhances independent agency. But unlike mobility, voluntary frontier settlement also emphasized the importance of the chosen destination to move. Sevincer compared students in a large city (Hamburg) and a smaller city (Braunschweig) and found that students who moved to the large city were more independent than those who either never moved or moved to a small city (Sevincer et al., 2015). This supported the idea that people with independent agency select into certain environments. In other words, besides the effect of mobility itself, certain environments are more likely to become concentrated with individualistic values.

One limitation of previous research is that it is hard to test whether historical events like voluntary settlement have really caused modern-day differences. Large-scale immigration to North America began in the 16th century and slowed down during the early 20th century due to the establishment of the immigration quotas. Westward frontier exploration and expansion took place mainly from the 17th century to the 19th century. Immigration to Hokkaido occurred mainly from the mid-19th century to the early 20th century. These natural cases make it difficult to fully control for potential confounding variables. That makes it difficult to pull apart self-selection from causal effects of the environment. Some scholars have argued that it is impossible to find frontiers to test in our modern, globalized world (Kitayama et al., 2014). However, we argue that the modern world does have frontiers that we can test in real time.

## Ongoing Voluntary Frontier

Are there any ongoing voluntary frontiers currently in existence that would be suitable for the examination of the voluntary settlement hypothesis? We argue that Shenzhen, China is an ideal location for this test.

In 1980, Shenzhen was a small town with only 30,000 people. In August of 1984, Shenzhen was designated as China’s first Special Economic Zone (SEZ). That meant it was selected as an experimental area for the practice of market capitalism. SEZs were set up primarily for agencies involved in foreign trade and factories with overseas capital and equipment. They offered preferential treatment to foreign firms seeking advantageous land use, rent agreements, and tax holidays (Chen, 1987; Chen and de’Medici, 2009).

Since 1980, millions of migrants voluntarily relocated to Shenzhen in search of better careers and economic opportunities provided by the city’s large-scale infrastructure construction and the large number of factory jobs (Chen and de’Medici, 2009). Shenzhen has become China’s largest city of migration. By 2016, the population had increased to 11.38 million people, with 3.55 million people having permanent *hukou* (Shenzhen Statistics Bureau, 2016). The *hukou* system is an important household registration system in use for the past 50 years in Mainland China. It is very important in people’s lives because numerous welfare benefits such as health insurance and education are determined based on different hukou categories, such as permanent hukou, blue-stamp hukou, or temporary residential permit. People who hold permanent and blue-stamp hukou in Shenzhen are those who relocated permanently to Shenzhen from their hometown. Approximately 70% of Shenzhen citizens do not have a permanent hukou, and the monthly turnover rate of Shenzhen’s population is approximately 25%, which means that 3 million people move in or out of Shenzhen every month (Li, 2011). Many people move in and out every year, coming from their hometowns to try to find opportunities in Shenzhen or leaving Shenzhen to go to other cities and countries (Ratti, 2003).

There are several reasons we think of Shenzhen as China’s “South Frontier”. First, “self-selection” was the main mechanism behind immigration to Shenzhen. Since the SEZ was established, millions of people—especially young people—voluntarily went to Shenzhen in pursuit of a better life. They were not forced to relocate; they decided by themselves to pursue an uncertain future by moving to a new city. Second, compared with other SEZ established at the same time, Shenzhen is the only SEZ that originally developed from a village into a first-tier metropolitan city. The other four SEZs were local cities in 1980s and are presently smaller than Shenzhen. Third, initially migrants were faced with a very challenging environment. Far away from friends, family, and familiar social support, migrants came to this city alone and needed to establish new social relationships. It required strong adaptability and mental strength to survive in an unfamiliar culture and environment. Fourth, after 30 years of rapid development and a large migrant population, Shenzhen has established its own unique image and culture with an atmosphere that is less politicized, free of many bureaucratic constraints, and replete with more varied economic opportunities (Chen, 1987). As an experimental field of reform, many new policies were intentionally tested in Shenzhen and then generalized to other mainland regions by the central government or unintentionally carried by migrants moving out of Shenzhen. This sort of social environment had a huge influence on newcomers, as well as child rearing practices. Fifth, Shenzhen is a Mandarin language zone. In the areas surrounding Shenzhen (and most of China) people usually speak dialects in their daily interactions. This is mainly because, in Shenzhen, people often do not know where the listener is from. Thus, it’s safe to use Mandarin Chinese, the national language. Sixth, there is some preliminary evidence that Shenzhen is an ongoing frontier. One study found that residents in Shenzhen showed more self-inflation on the sociogram task, a measure of implicit individualism (Chen et al., 2016). Shenzhen residents also showed more attention to the focal object in the Framed Line Task, a measure of analytic perceptual style.

Overall, Shenzhen appears to be an ongoing voluntary frontier settlement providing opportunities to explore the relationship between voluntary frontier settlement and independent agency and its underlying mechanisms directly. In this study, we hypothesize that, even with such a short migration history and an ongoing voluntary settlement process, voluntary frontier settlement will lead Shenzhen to score higher on independent agency than other parts of China. In other words, residents in Shenzhen will be more independent than people who have remained in their hometowns. Of course, voluntary settlement is not the only feature of Shenzhen. To test competing hypotheses, we compare people in Shenzhen to a similarly large, developed city. We also controlled for relational mobility.

## Method

### Participants

Two hundred and twelve adults from Shenzhen (64 men, 148 women, *M*age = 29.6 years) and one hundred and two adults from Wuhan (29 men, 73 women, *M*age = 33.0 years) completed the study online. Questionnaires were put on the website www.sojump.com, and the participants submitted their answers via cell phones or computers. On average 20 min were needed to finish each questionnaire. All the participants were recruited by researchers using social networks and snowball sampling techniques.

It should be kept in mind that it is impossible to find a perfect counterpart city—a city that is similar on all potential confounding factors but is not a frontier like Shenzhen. Initially, we used several indicators to select a counterpart city: population size, economic indicators such as GDP per capita, contact with Western culture, and the cosmopolitan index (Sevincer et al., 2015, 2017). Based on this, we created a list of candidate cities. Next, we compared the strengths and weaknesses of these cities in their comparability to Shenzhen.

After looking at these indicators, we chose Wuhan for several reasons. First, the population of Wuhan is about 10 million, which is very similar to Shenzhen. Second, GDP per capita of Wuhan is 104,132 Yuan, which is higher than most cities in China, although it is lower than Shenzhen (157,985). Third, based on Sevincer’s cosmopolitan city index which was measured by eight items such as “multicultural city” and “center for science and research” on a 7-point scale range from 1(not at all) to 7 (very much), Shenzhen scores 6.6 while Wuhan scores 6.2.

### Materials

#### Dependent Variables

**Independent and interdependent self**: We measured independence/interdependence using the Singelis Self-Construal Scale (Singelis, 1994). The scale has 12 interdependent items and 12 independent items that are mixed and randomly ordered. Respondents rated their agreement with the items on a scale from 1 (strongly disagree) to 7 (strongly agree). Interdependent items generally ask about people’s interpersonal interactions, such as “it is important for me to maintain harmony within my group” and “I will sacrifice my self-interest for the benefit of the group I am in”. Independent items describe individuals’ observations of the self, with items including “I act the same way no matter who I am with” and “being able to take care of myself is primary concern for me”. In this study, Cronbach alphas were 0.83 for both the independent and interdependent subscales.

**Loyalty/Nepotism:** We measured the loyalty/nepotism, also called the reward and punishment task (Wang et al., 2011). The loyalty/nepotism task has been used to measure in-group favoritism. In the task, participants read about a friend or stranger who behaved dishonestly or honestly. In the friend conditions, participants were asked to choose one of their best friends and write down their name. The honest friend condition reads:

You and your friend recently completed a business deal and you have just discovered that he/she was honest about some key information. As a result, you received $1,000. You would have received 50% less if he/she had been dishonest.

Thus, in the honest condition, participants expected $500 and received $1,000. In the deception condition, participants received $1,000 because the friend was dishonest, but had expected a $1,500 gain if the friend had been honest. Respondents could then spend hypothetical money to reward (in the honest condition) or punish (in the dishonest condition) this friend at a cost. The cost was set at one tenth of the reward or punishment amount. Punishment and reward amounts and their costs were presented on 11-point scales from $0 to $100, in $10 increments. For example, participants could punish the person $100 at a cost of $10.

A prior study found that participants in Singapore showed stronger favoritism toward their friends than Americans did (Wang et al., 2011). Singaporeans rewarded friends more than they punished them, but they did not show this favoritism toward strangers. This task was also used as measurement of social style comparing northern wheat-farming parts of China versus southern rice-farming parts of China (Talhelm et al., 2014).

#### Independent Variable

**Frontier**: The independent variable was frontier environment (Shenzhen) versus non-frontier (Wuhan).

#### Mediating Variable

**Relational mobility**: We measured relational mobility using Yuki et al. (2013) 12-item scale. The scale asks participants to report their perceptions of relational mobility for people in their immediate environment from 1 (*strongly disagree*) to 6 (*strongly agree*). Relational mobility is the degree to which individuals in a society have opportunities to form new relationships and terminate old ones (Schug et al., 2010). Examples sentences include: “They can choose who they interact with” and “It is easy for them to make new friends”. In this study, the Cronbach alpha reliability for the relational mobility scale was 0.79.

#### Demographic Variables

We included several demographic variables: age, gender, subjective socioeconomic status, parental educational attainment, and household monthly income. Subjective socioeconomic status was rated by one item “people sometimes describe themselves as belonging to some class below, would you describe yourself belonging to the _”. Participants rated their social status on a 5-point scale: 1 = lower class, 2 = lower middle class, 3 = middle class, 4 = upper middle class, 5 = upper class. Participants reported their household monthly income on a 10-point scale from 1 (less than 1,000 Yuan) to 10 (more than 50,000 Yuan). Participants rated their parents’ educational attainment (taking the higher of their parents’ educational attainment) from 1 (primary school or less) to 6 (graduate school or higher).

## Results

Initially, we explored whether or not the two groups were well controlled on demographic statistics. We found no significant differences on gender, χ_(1)_^2^ = 0.1, *p* = 0.75; subjective socioeconomic status (*M*_shenzhen_ = 3.48, *SD*_shenzhen_ = 0.68; *M*_wuhan_ = 3.50, *SD*_wuhan_ = 0.74), *t*(312) = 0.28, *p* = 0.78; parents’ educational attainment (*M*_shenzhen_ = 3.03, *SD*_shenzhen_ = 1.14; *M*_wuhan_ = 3.04, *SD*_wuhan_ = 1.09), *t*(312) = 0.08, *p* = 0.94. However, Shenzhen residents had higher household incomes (*M*_shenzhen_ = 6.47, *SD*_shenzhen_ = 2.02) than Wuhan residents (*M*_wuhan_ = 4.73, *SD*_wuhan_ = 2.41), *t*(312) = 6.73, *p* < 0.01; the Shenzhen residents were older (*M*_shenzhen_ = 33.0, *SD*_shenzhen_ = 5.13) than Wuhan residents (*M*_wuhan_ = 29.6, *SD*_wuhan_ = 8.86), *t*(312) = 3.35, *p* < 0.01.

### Explicit Beliefs of Independent/Interdependent Self

The descriptive statistics of independent and interdependent scores were reported in **Table 1**. The scores were submitted to a 2 (self: interdependent vs. independent) × 2 (frontiers: Shenzhen vs. Wuhan) ANOVA. The frontier was considered a between-subjects factor and self was considered a within-subjects factor.

**Table 1:** Descriptive statistics of independent and dependent variables by two groups.

	Shenzhen Mean (*SD*)	Wuhan Mean (*SD*)	*P*
Inds	4.77 (0.64)	4.54 (0.78)	0.006^∗∗^
Inters	5.04 (0.68)	5.11 (0.77)	0.45
RM	4.33 (0.59)	4.09 (0.53)	<0.001^∗∗∗^
RS	29.39 (48.34)	13.73 (42.56)	0.004^∗∗^
RF	56.42 (43.37)	50.10 (44.51)	0.23
*N*	212	102	

*Inds, Independent self; Inters, Interdependent self; RM, Relational mobility; RF, Reward friends; RS, Reward strangers. ^∗^*p* < 0.05; ^∗∗^*p* < 0.01; ^∗∗∗^*p* < 0.001.*

The main effect of frontier was not significant, *F*(1,312) = 1.25, *p* = 0.26. However, the interaction of self and frontier was significant, *F*(1,312) = 12.14, *p* = 0.001, $\eta_{p}^{2}$ = 0.037. For independent self, Shenzhen (*M* = 4.77, *SD* = 0.64) was higher than Wuhan (*M* = 4.54, *SD* = 0.78), *t*(312) = 2.76, *p* = 0.006, *d* = 0.33. For interdependent self, there was no significant differences between Wuhan (*M* = 5.11, *SD* = 0.77) and Shenzhen (*M* = 5.04, *SD* = 0.68), *t*(312) = 0.76, *p* = 0.45, *d* = 0.09.

To investigate whether Shenzhen-Wuhan differences remained robust when controlling for the potential confounding effect of demographic variables, we first dummy-coded participants who lived in Shenzhen and Wuhan (frontier: Wuhan = 0, Shenzhen = 1) and then conducted a regression analysis. The results were reported in **Table 2**. With reference to independent self, after controlling for age, gender, household monthly income, parental educational attainment and subjective SES, frontier still positively predicted independent self, β = 0.17, *t*(312) = 2.78, *p* = 0.006. With reference to interdependent self, after controlling for age, gender, household monthly income, parental educational attainment and subjective SES, frontier did not predict interdependent self, β = 0.002, *t*(312) = 0.04, *p* = 0.97.

**Table 2:** Summary of regression predicting independent/interdependent self and reward friend/stranger and relational mobility as a function of Demographic and Relational mobility and Voluntary Frontier Settlement.

	Independent self		Interdependent self		Reward friend		Reward stranger		Relational mobility	
	Beta	Δ*R*^2^	Beta	Δ*R*^2^	Beta	Δ*R*^2^	Beta	Δ*R*^2^	Beta	Δ*R*^2^
Age	0.17ˆ**	0.046ˆ*	0.10	0.057ˆ**	0.12ˆ*	0.027	0.09	0.019	0.09	0.031
Gender	–0.10		–0.12ˆ*		–0.11		–0.09		0.04	
SSES	0.01		–0.02		–0.03		0.02		–0.05	
MHI	–0.14ˆ*		–0.19ˆ**		–0.01		–0.10		0.04	
PE	0.12		0.18ˆ**		–0.03		0.03		0.09	
Frontier	0.17ˆ*	0.024ˆ***	0.00	0.000	0.05	0.002	0.17ˆ**	0.026ˆ*	0.19ˆ**	0.03ˆ**

*MHI, Monthly household income; SSES, Subjective socioeconomic status; PE, parental educational attainment; RM, Relational mobility; ^∗^*p* < 0.05; ^∗∗^*p* < 0.01; ^∗∗∗^*p* < 0.001.*

### Relational Mobility

The relational mobility scores are reported in **Table 1**. The scores between the two groups were compared with independent-sample *t*-test. Shenzhen group scores (*M* = 4.33, *SD* = 0.59) were significantly higher than Wuhan group scores (*M* = 4.09, *SD* = 0.53), *t*(219) = 3.76, *p* = 0.000, *d* = 0.54.

To investigate whether or not Shenzhen-Wuhan differences remained robust when controlling the potential confounding effect of gender and social economic status, we first dummy-coded participants who lived in Shenzhen and Wuhan (frontier: Wuhan = 0, Shenzhen = 1) and then the regression analysis was done. For relational mobility, when age, gender, household monthly income, parental educational attainment and subjective SES were controlled, frontier still positively predicted relational mobility, β = 0.19, *t*(312) = 3.14, *p* = 0.002.

### Nepotism

The descriptive statistics of reward and punishment scores were reported in **Table 1**. The scores were submitted to a 2 (nepotism: reward friends vs. reward strangers) × 2(frontiers: Shenzhen vs. Wuhan) ANOVA. The frontier was considered the between-subjects factor and nepotism within-subjects factor.

The main effect of frontier was statistically significant, *F*(1,312) = 5.46, *p* = 0.02, $\eta_{p}^{2}$ = 0.017. Shenzhen (*M* = 42.90, *SD* = 45.52) was higher than Wuhan (*M* = 31.91, *SD* = 43.53).

The interaction between nepotism and frontier was marginally significant, *F*(1,312) = 2.91, *p* = 0.09, $\eta_{p}^{2}$ = 0.009. For rewarding strangers, Shenzhen scored (*M* = 29.39, *SD* = 48.34) higher than Wuhan (*M* = 13.73, *SD* = 43.56), *t*(312) = 2.92, *p* = 0.004, *d* = 0.35. For rewarding friends, there were no differences between Shenzhen (*M* = 56.42, *SD* = 43.37) and Wuhan (*M* = 50.10, *SD* = 44.51), *t*(312) = 1.20, *p* = 0.23, *d* = 0.15.

To investigate whether Shenzhen-Wuhan differences remained robust when controlling for the potentially confounding effect of demographic variables and relational mobility, we first dummy-coded participants who lived in Shenzhen and Wuhan (frontier: Wuhan = 0, Shenzhen = 1) and then the regression analysis was done. The results were reported in **Table 2**. With reference to rewarding a friend, after controlling for age, gender, household monthly income, parental educational attainment and subjective SES, frontier didn’t predict rewarding friend, β = 0.05, *t*(312) = 0.74, *p* = 0.46. With reference to rewarding a stranger, after controlling for age, gender, household monthly income, parental educational attainment and subjective SES, frontier still predicted rewarding stranger, β = 0.17, *t*(312) = 2.85, *p* = 0.005.

### Mediation Analysis

**FIGURE 1:**
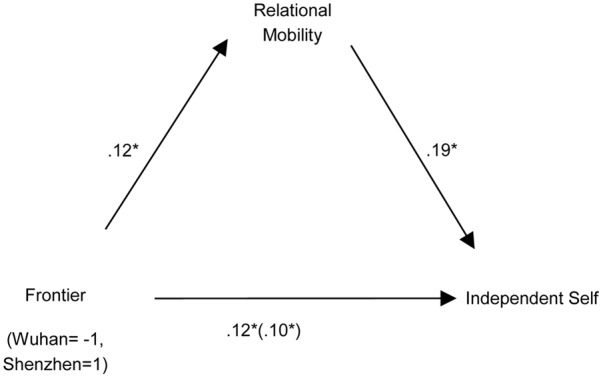
Unstandardized betas from a path analysis examining the role that relational mobility plays in mediating the effects of Frontier and independent self. ^∗^*p* < 0.05.

We conducted a mediation analysis to examine mobility as alternative explanation. We followed Shrout and Bolger’s (2002) procedure. For independent self, we tested a mediation model (**Figure 1**) that included frontier (Shenzhen vs. Wuhan), relational mobility, and independent self. Frontier predict relational mobility, *B* = 0.12, β = 0.19, *F*(1,312) = 3.32, *p* = 0.002; relational mobility predicted independent self, *B* = 0.22, β = 0.19, *F*(1,312) = 4.45, *p* = 0.004. Relational mobility partially mediated the relationship between frontier and independent self, Sobel *z* = 2.29, *p* = 0.016 (**Figure 1**). The meditation effect of relational mobility accounted for 17.8% of total effect of frontier on independent self. No other mediation effects were found.

The results implied that relational mobility can partially mediate regional differences between Wuhan and Shenzhen on independent self, and, at the same time, most of the variance in independent self could be explained by voluntary frontier settlement. However, relational mobility seemed to have no effect on rewarding a stranger.

### Length of Stay in Shenzhen

For Shenzhen residents, correlation analysis was employed to explore whether the length of the stay in Shenzhen predicted three individualistic measures. The results were reported in **Table 3**. Length of stay in Shenzhen was not related to independent/interdependent self, relational mobility or nepotism. As about three individualistic measurement, relational mobility was positively related to independent self, *r*(212) = 0.19, *p* = 0.006; independent self was positively related to interdependent self, *r*(212) = 0.45, *p* < 0.001; reward friends was positively related to reward strangers, *r*(212) = 0.50, *p* < 0.001. Other correlations were not significant.

**Table 3:** Descriptive statistics and correlation results of length of stay in Shenzhen and individualistic variables.

	*M* (*SD*)	1	2	3	4	5	6
(1) Inds	5.04 (0.68)						
(2) Inters	4.77 (0.64)	0.45^∗∗∗^					
(3) RM	4.33 (0.59)	0.19^∗∗^	0.05				
(4) RF	56.42 (43.37)	–0.01	0.02	0.11			
(5) RS	29.39 (48.34)	0.06	–0.01	0.08	0.50^∗∗∗^		
(6) LS	10.42 (6.21)	0.10	0.05	0.08	0.02	–0.08	

*Inds, Independent self; Inters, Interdependent self; RM, Relational mobility; RF, Reward friends; RS, Reward strangers; LS, Length of stay in Shenzhen. ^∗∗∗^*p* < 0.001; ^∗∗^*p* < 0.01.*

## General Discussion

This study focused on Shenzhen, an ongoing frontier settlement in South China that started growing in the 1980s and has continued to grow for nearly four decades. We tested the hypothesis that a place at the early stage of voluntary frontier settlement will have higher independent agency. As predicted, on two different measures of independent agency, Shenzhen residents were more independent than people in Wuhan. On self-reported explicit beliefs, Shenzhen residents reported being independent than Wuhan residents, while there were no differences on interdependent self. For loyalty/nepotism, Shenzhen residents rewarded strangers more than Wuhan residents, while there were no differences in rewarding friends. Furthermore, even after controlling for relational mobility, frontier differences still remained on independent agency, although the effects decreased in some aspects such as independent self.

Finally, length of stay in Shenzhen had no effect on independent agency. This could imply that self-selection is the mechanism of voluntary frontier settlement, at least in Shenzhen. However, more detailed comparisons are needed to tease apart the effect of the environment in Shenzhen versus self-selection.

### Voluntary Frontier Settlement and Mobility Hypothesis

This study found that voluntary frontier settlement predicted independent agency even after controlling for other confounding factors. This study controlled for some variables that prior researches on voluntary frontier settlement and mobility have not controlled for. For example, researches on mobility have not parsed out the effect of moving from the effect of moving to specific locations (like a frontier city). The concept of relational mobility has come out more recently and thus has not been tested in frontier research (Kitayama et al., 2006, 2009; Ishii, 2014; Ishii et al., 2014). One study on the cosmopolitan city hypothesis found that people’s motivation toward independence affected their residential choices—that is, independent people were more likely to move to cosmopolitan cities (Sevincer et al., 2015).

In this study, we controlled for one type of mobility, relational mobility. Frontier settlement still predicted independent self in explicit belief and reward toward stranger in nepotism task. Relational mobility partially mediated the relationship between frontiers (Shenzhen vs. Wuhan) and independent beliefs. Relational mobility could explain why Shenzhen residents are more independent than Wuhan in part. It implied that there are multiple antecedents of independent agency. These results suggest that we need to continue to search for more variables to explain more variance in individualism.

This study also brings more nuances to the idea of urban/rural differences. The city air hypothesis talks about urban-rural differences in social constraint. Yet Wuhan is another big city, similar to Shenzhen in size. Urban/rural differences alone cannot account for the differences between Shenzhen and Wuhan. We argue that there are other cultural differences between cities beyond size and development.

### Self-selection and Adaption Mechanism

Another contribution of this paper was to explore relative importance of underlying mechanisms in early stage of voluntary frontier settlement. Self-selection and adaption are two psychological processes related to frontier settlement (Kitayama et al., 2006). The self-selection mechanism holds that highly independent individuals are more likely to immigrate to Shenzhen to pursue personal wealth. The adaptation mechanism holds that Shenzhen has an environment of individualism, so people who move there become more individualistic over time. People’s length of the stay in Shenzhen was not correlated to explicit beliefs or nepotism, which fits more with the self-selection mechanism. This finding is consistent with some findings in previous research. For example, Kitayama found that self-selection operated primarily on psychological process toward personal choice and personal goal pursuit (Kitayama et al., 2006, Studies 1 and 2). Sevincer found that self-selection was involved in cosmopolitan settlement in self-reported priming conditions (Sevincer et al., 2015). Thus, self-selection seems to play an important role in frontier culture.

### Interdependent Agency in Ongoing Frontier

It was also found that there were no differences on interdependent self in self-reported explicit beliefs and reward toward friends. At first glance, the results did not match the hypothesis where Shenzhen residents should score lower on interdependent self and reward friend compared to Wuhan counterpart. However, it is possible there are only differences on independent self and at the same time no differences on interdependent self between Shenzhen and Wuhan. Theoretically, both independent and interdependent self were needed when individuals grew up for their adaption toward social and cultural environment. In Shenzhen, the independent self was enhanced only in some domains, especially for personal success and striving for wealth. But in other domains such as family harmony interdependent self was helpful to maintain self-identity and there’s no pressure to be changed. It was only under some circumstance when there’s inner conflict between independent and interdependent self, independent self would replace interdependent self through chronic practice. For example, during holidays, one might have to make decision to either have a trip alone or to go home to visit parents. Such as in nepotism task of this study, the main differences observed between Wuhan and Shenzhen were toward strangers and there were no differences toward friends. This furthermore suggested that social ecology of Shenzhen led to adaptive strategy where it was better to reward stranger rather than punish him for his interest while at the same time it was not necessary to change the attitude toward friends. It just implies that it is more common to have to deal with strangers in Shenzhen than in Wuhan. Secondly, some evidences showed that this pattern was possible. A few studies examined the cultural changing in the past decades in China. Using multiple individualism and collectivism word index from Google Library Viewer and Chinese Corpus, scholars found that since 1970s individualism increased quickly while at the same time collectivism didn’t change or it increased more slowly in contrast with individualist values (Zeng and Greenfield, 2015).

As in prior research, there were very weak correlations or no correlations between the different cultural tasks (Kitayama et al., 2009; Na et al., 2010). These tasks show coherence at the group level, but not always at the individual level (for a more detailed statistical explanation of group-level versus individual-level constructs, see Na et al., 2010).

### Limitations and Future Directions

We should note several limitations in this study. First, we did not follow Kitayama’s triangulation method to test the voluntary frontier settlement hypothesis that one group from a western society should be included (Kitayama et al., 2006). Future studies should include one group from western society to come up with more robust conclusions. Second, we used only a few cultural tasks. Including other cultural tasks would be a more robust test (such as the Framed Line Task and the Triad Task, as well as cultural products such as percentage of popular baby names).

The third was the variation and direction of voluntary frontier settlement on independent agency. Unlike frontier settlement in United States which is surrounded by the whole independent culture, it seemed that original culture and frontier ethos promoted mutually and that would help to maintain and exert its effect on frontier and its offspring’s independent mind and behavior. Shenzhen was surrounded by interdependent culture that means the innate conflicts between frontier independent ethos and original interdependent culture would obstruct the frontier’s impact on mind and behavior in the long term. In actual, some scholars also pointed out that frontier’s independent ethos might fade and finally disappear in Hokkaido which frontier was also surrounded by interdependent culture (Ishii et al., 2014).

The fourth was which form of independent agency would be strengthened mainly in Shenzhen’s ongoing frontier settlement. It was suggested that different initial conditions shed light on the relationship between voluntary frontier settlement and different forms of independent agency. In the United States with the prior condition of independence, motivational independence was emphasized which is strong enough to influence all aspects of life. In Japan with the prior condition of interdependence normative independence was emphasized and strong enough to avoid risk of violating these norms (Kitayama and Bowman, 2010; Ishii, 2014). Ishii found in Hokkaido that when norm was primed by schematic faces, Hokkaido residents showed vocal stroop interference effect similar to United States and different from mainland Japanese. These finding supported the above presented idea (Ishii, 2014). Whether Shenzhen belongs to one of these or some third form of independent agency still needs to be studied in future.

The fifth was about possible biological mechanism of voluntary frontier settlement. For example, self-selection was suggested as behavior mechanism of the hypothesis. Furthermore, self-selection was related to certain genetic factors such as DRD4 and 5HTTLPR (Chiao and Blizinsky, 2009; Kitayama et al., 2014). Shenzhen as an ongoing frontier would give us a perfect environment to test whether or not there’re some specific biological mechanism underlying voluntary frontier settlement in the future.

Finally, this study has the limitation that it only compared two cities that have many differences besides being a frontier city. One way to make up for this limitation would be to include more comparison cities. In this study, Wuhan was selected not arbitrarily, but deliberately with several societal indicators similar to Shenzhen. However, there is no perfect contrast group for this kind of natural comparison. But it could be well improved when a few metropolitan cities were included as multiple contrast groups in one study in future.

## Conclusion

The present work is one of the first attempts to directly examine the voluntary frontier settlement hypothesis using ongoing voluntary frontier settlement and that self-selection mechanism in early stage might play more important role than other mechanisms. Our results suggested that, even in an interdependent culture, ongoing voluntary frontier settlement leads to independent agency.

## Ethics Statement

This study was carried out in accordance with the recommendations of Institutional Review Board of the Institute of Psychology, Chinese Academy of Sciences with written informed consent from all subjects. All subjects gave written informed consent in accordance with the Declaration of Helsinki. The protocol was approved by the Institutional Review Board of the Institute of Psychology, Chinese Academy of Sciences.

## Author Contributions

JF made substantial contributions to the acquisition of data for the work and the design of the work. XR made substantial contributions to the conception and design of the work and drafting the manuscript. XM made substantial contributions to the analysis and interpretation of data for the work.

## Conflict of Interest Statement

The authors declare that the research was conducted in the absence of any commercial or financial relationships that could be construed as a potential conflict of interest.
